# Getting to know adenosine deaminase 2 deficiency inside and out

**DOI:** 10.1016/j.jaci.2025.01.040

**Published:** 2025-05

**Authors:** Lisa Ehlers, Isabelle Meyts

**Affiliations:** a Department of Microbiology, Immunology and Transplantation, Laboratory for Inborn Errors of Immunity, Katholieke Universiteit (KU) Leuven, Leuven, Belgium; b Department of Pediatric Respiratory Medicine, Immunology and Critical Care Medicine, Charité–Universitätsmedizin Berlin, corporate member of Freie Universität Berlin and Humboldt-Universität zu Berlin, Berlin, Germany; c Berlin Institute of Health at Charité–Universitätsmedizin Berlin, Berlin, Germany; d German Center for Child and Adolescent Health (DZKJ), partner site Berlin, Berlin, Germany; e Deutsches Rheuma-Forschungszentrum, an Institute of the Leibniz Association, Berlin, Germany; f Department of Pediatrics, University Hospitals Leuven, KU Leuven, Leuven, Belgium

**Keywords:** Adenosine deaminase 2, deficiency of ADA2, autoinflammation, immunodeficiency, inborn errors of immunity, type I interferons

## Abstract

Ten years after the description of the first cohorts of patients with adenosine deaminase (ADA2) deficiency (DADA2), the pathomechanisms underlying the disease on a cellular level remain poorly understood. With the establishment of the lysosomal localization of the ADA2 protein and its involvement in nucleic acid sensing, the pathophysiologic focus has shifted to the inside of the cell. At the same time, extracellular (serum) ADA2 enzyme activity continues to be the diagnostic reference standard in patients with suspected DADA2. The diverse clinical phenotype and weak genotype–phenotype correlations further complicate the identification of shared cellular mechanisms that cause inflammation, immunodeficiency, and bone marrow failure in the absence of functional ADA2. This review inspects the characteristics of the ADA2 protein and its proposed function. The latter is discussed in the context of possible mechanisms driving the clinical phenotype in patients lacking functional ADA2. We discuss established processes and introduce unexplored pathways in the pathogenesis of DADA2.

## Adenosine deaminase 2

### Identification of a second human adenosine deaminase

In 1978, researchers first described residual adenosine deaminase activity in the tissue of patients with severe combined immunodeficiency due to deleterious mutations in *ADA,* encoding for human adenosine deaminase (hereafter referred to as ADA1).^1^^,^^2^ The protein responsible for the deamination of adenosine in the absence of functional ADA1 accounted for 1-2% of adenosine deaminase activity in the spleen tissue of healthy controls. The characteristics of this second human adenosine deaminase differed from ADA1 in several ways. The enzyme—now known as adenosine deaminase 2 (ADA2)—was found to have a (1) higher molecular weight, (2) lower pH optimum at pH 6.5, and (3) higher Michaelis-Menten constant (*K*_*m*_). ADA2 was not inhibited by erythro-9-(2-hydroxy-3-nonyl)adenine (EHNA), an efficient inhibitor of ADA1. In subsequent years, ADA2 was found to be encoded by a gene in the region of the long arm of chromosome 22 that is duplicated in patients with cat eye syndrome and was therefore initially named cat eye syndrome critical region protein 1 (*CECR1*).^3^

ADA2 is primarily expressed in immune cells with monocytes, macrophages, and dendritic cells showing the highest protein expression.^4^ The protein is secreted to the extracellular space, and elevated levels of ADA2 have been found—for example, in plasma or pleural effusions in the context of inflammation, infection, or cancer.^5^, ^6^, ^7^, ^8^ Examples of diseases that are characterized by increased ADA2 levels include tuberculosis, systemic lupus erythematosus, and macrophage activation syndrome.^9^, ^10^, ^11^ Moreover, it has been shown that in children, plasma ADA2 enzyme activity is negatively correlated with age.^12^

Both ADA1 and ADA2 metabolize adenosine and 2′-deoxyadenosine to inosine and 2′-deoxyinosine, respectively. Compared to ADA1 (*K*_*m*_ = 40 μmol), ADA2 has a more than 50-fold lower affinity for adenosine (*K*_*m*_ = 2.5 mmol) and is inferior at deaminating deoxyadenosine as opposed to adenosine.^13^, ^14^, ^15^ Although ADA2 is usually referred to as the *extracellular* human adenosine deaminase, ADA2 accounted for the majority of the intracellular adenosine deaminase activity of dendritic cells.^16^ Despite catalyzing the same enzymatic reaction, the functions of ADA1 and ADA2 in the human body are not redundant. This is evident from the severe clinical phenotypes of patients lacking either enzyme: ADA1 deficiency causes severe lymphopenia (ADA–severe combined immunodeficiency) as a result of intracellular accumulation of toxic purine metabolites.^17^ Deficiency of ADA2 (DADA2), in contrast, causes a complex phenotype of autoinflammation and immunodeficiency.^18^

### Characteristics of the ADA2 protein

ADA1 is a 42 kDa monomeric enzyme that resides in the cytoplasm but can localize to the cell surface bound to the ADA-binding protein dipeptidyl peptidase IV/CD26.^14^ Although the catalytic domains of the two adenosine deaminases are structurally very similar,^19^ the *ADA* and *ADA2* genes are not homologous. The protein sequence of ADA2 was found to strongly resemble that of a group of proteins referred to as adenosine deaminase–related growth factors expressed by a number of insects and vertebrates, including fruit fly, zebrafish, frog, chicken, and pig, but absent in rodents.^3^^,^^20^^,^^21^ These are secretory proteins that contain an ADA-like catalytic domain in the C-terminal part. Their growth factor function is mediated by their adenosine deaminase activity.^21^ Compared to its evolutionarily early orthologs expressed in insects like *Lutzomyia longipalpis* (*K*_*m*_ = 15 μmol) or *Sarcophaga peregrina* (*K*_*m*_ = 50 μmol), human ADA2 shows reduced affinity to its substrate adenosine (*K*_*m*_ = 2.5 mmol) as a result of the increased hydrophilicity of the binding pocket.^22^, ^23^, ^24^ The evolution of the ADA2 protein toward a lower affinity for adenosine and the fact that ADA1—a highly efficient adenosine deaminase universally expressed in human immune cells—does not compensate for its absence suggest that ADA2 has assumed a different protein function in human cells or is specifically responsible for the regulation of purine metabolites at sites of high adenosine concentration.

**Fig 1:**
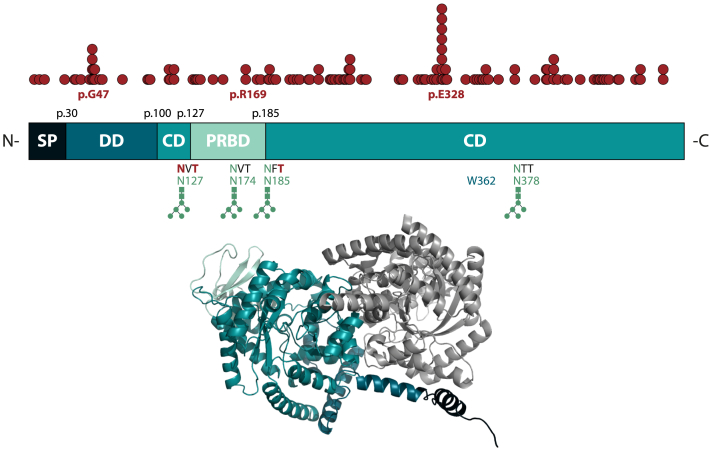
Protein structure of ADA2. Different protein domains are highlighted in *different colors,* with the second unit of the homodimer depicted in *gray. N*-Glycosylation sites are indicated in *green. Top, Red dots* depict all pathogenic *ADA2* variants reported to date at their corresponding location across the amino acid sequence. Variants involving glycosylation sites are shown *below,* with the affected amino acid highlighted in *red. Bottom,* 3D structure created with PyMOL v3 after importing structural information from AlphaFold.^107^*CD,* Catalytic domain; *DD,* dimerization domain; *PRBD,* putative receptor binding domain; *SP,* signal peptide.

The *ADA2* gene comprises 10 exons encoding a 511 amino acid protein.^25^ Each ADA2 monomer has a molecular weight of 60 kDa. The protein is composed of 4 domains that reflect several characteristics of the protein (Fig 1).^19^ The N-terminal signal peptide mediates secretion of ADA2 via the secretory pathway. The dimerization domain forms a hydrophobic area of α helices that enables the interaction of the 2 subunits of the dimer around a central tryptophan residue (p.W362). The C-terminal catalytic domain represents the largest part of the protein and—besides containing the active site—also contributes to the dimerizing surfaces. Within the catalytic domain, the putative receptor binding domain forms a disulfide bond that is indispensable for protein secretion. This area of the protein also harbors 3 of the 4 *N*-glycosylation sites of ADA2 (p.N127, p.N174, p.N185). Another one is located in the catalytic domain (p.N378). The glycan structures make up approximately 6 kDa of the molecular weight of the monomer.^13^ *N*-Glycosylation is required for folding, secretion, and activity of ADA2.^26^ Genetic variants affecting one of the *N*-glycosylation sites lead to accumulation of the resultant protein in the endoplasmic reticulum (ER) and the intracellular formation of protein aggregates.^26^^,^^27^

ADA2 binds to glycosaminoglycans and proteoglycans like heparin and immunoglobulins but also glycan structures on the cell surface.^16^^,^^24^ Among immune cells, neutrophils, monocytes, B cells, and natural killer cells show the strongest surface binding of ADA2.^28^ The protein also binds to CD39^+^ regulatory T cells. Contrary to the other cells, the structures responsible for the attachment of ADA2 to the T-cell surface have been proposed to be the adenosine receptors A2a and A2b.^16^ This binding is thought to regulate the receptors’ affinity to their natural ligands and thus the activity of the downstream anti-inflammatory pathways. Moreover, the degradation of adenosine by extracellular ADA2 has been shown to increase monocytic TNF-α secretion, most likely by counteracting the adenosine-induced suppression of cytokine secretion mediated by binding to adenosine receptor A2a.^28^ Unlike the growth factor effect described in, for example, insects and frogs, ADA2 induces proliferation of CD4^+^ T cells independently of its deaminase activity. This requires the direct interaction of the T cells with monocytes. In addition, the addition of ADA2 to peripheral blood mononuclear cells in coculture induces the differentiation of monocytes into macrophages.^16^

### Extracellular purine metabolism

**Fig 2:**
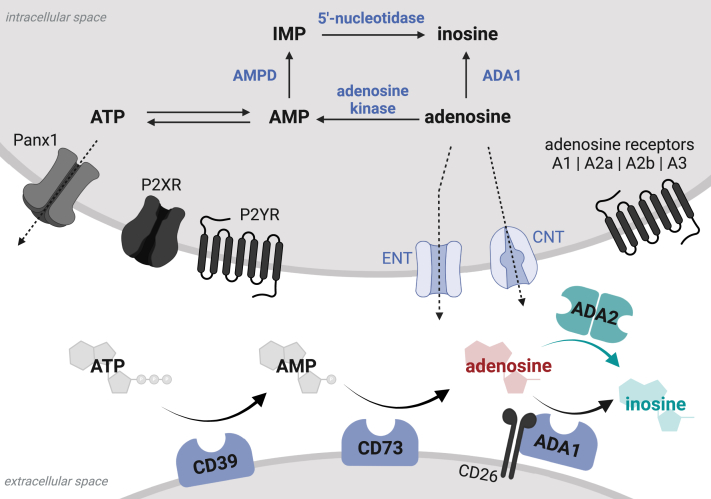
Extracellular purine metabolism. ADA2 mediates the extracellular deamination of adenosine as part of an ensemble of ectoenzymes. *AMPD,* AMP deaminase; *CNT,* concentrative nucleoside transporter; *ENT,* equilibrative nucleoside transporter; *P2XR,* P2X purinoreceptor; *P2YR,* P2Y purinoreceptor; *Panx1,* pannexin 1. Created with BioRender.com.

The extracellular purine metabolism plays a crucial role in the regulation of the inflammatory environment. For simplification, this complex interplay of metabolites can be broken down into the balance between proinflammatory ATP and anti-inflammatory adenosine. Extracellular purine levels are orchestrated by a number of ectoenzymes and channels (Fig 2): the subsequent reactions of ectonucleoside triphosphate diphosphohydrolase 1 (CD39) and 5′-nucleotidase (CD73) metabolize ATP to AMP and then to adenosine, thereby having an overall anti-inflammatory effect.^29^^,^^30^ The action of extracellular adenosine deaminases, in contrast, is proinflammatory, as they deaminate adenosine and thus increase the extracellular ATP:adenosine ratio.^31^ Next to the ectoenzymes, channel proteins and transporters affect the concentrations of extracellular purine metabolites by allowing an exchange with the intracellular milieu. On the one hand, ATP is transported across the membrane via pannexin 1 and connexin 43.^32^^,^^33^ Adenosine levels, on the other hand, are adjusted via equilibrative and concentrative nucleoside transporters.^34^

The effects of extracellular adenosine are mediated by the G protein–coupled adenosine receptors A1, A2a, A2b, and A3. The A2a adenosine receptor is highly expressed on human immune cells, and its activation causes strong anti-inflammatory effects.^35^^,^^36^

In the absence of ADA2, we would therefore expect a surplus of extracellular adenosine leading to an overall anti-inflammatory environment. Consequently, the inflammatory phenotype observed in DADA2 patients constitutes an apparent discrepancy and highlights the need for a more detailed understanding of the role of ADA2 in the regulation of extracellular adenosine metabolites.

### Function of the ADA2 protein

While the ADA2 protein has the capacity to deaminate adenosine, it remains a matter of debate whether adenosine deamination is the only or the most relevant function of the protein. ADA2 is often referred to as the main adenosine deaminase in human serum. This is based on experiments performed at saturating concentrations of adenosine for ADA2 equivalent to >10,000 times physiologic plasma adenosine concentrations.^37^ ADA1 is, however, also found extracellularly as ecto-ADA1 bound to CD26, and plasma ADA1 activity has been reported to be about 5 U/L.^38^ Given the difference in affinity for adenosine between ADA1 and ADA2 at physiologic adenosine levels of 10-20 nmol/L in human plasma, the amount of adenosine metabolized by ADA2 should be almost negligible. Recently, researchers showed that—transfected into HEK293T cells—only 2% of ADA1 was found in the supernatant while ADA2 was completely secreted. Even under these conditions, extracellular adenosine deamination by ADA1 surpassed that of ADA2.^39^ Zavialov et al therefore proposed that ADA2 regulates adenosine levels at sites of inflammation.^19^ The inflammatory milieu can be characterized by higher concentrations of adenosine and a lower pH, in line with the enzyme characteristics of ADA2. Tiwari-Heckler et al showed that ADA2 can induce a proinflammatory and profibrotic phenotype (corresponding to M2b polarization) in human monocyte–derived macrophages *in vitro*. These changes did not occur upon incubation with mutant ADA2 lacking adenosine deaminase activity.^40^

Indeed, the facts that ecto-ADA1 expression is also upregulated under inflammatory conditions and that human ADA2 has evolved toward reduced affinity for adenosine suggest that the nonredundant function of the ADA2 protein in the human body is not adenosine deamination.^19^^,^^41^ Recently, an alternative intracellular function of ADA2 has been proposed. In 2020, Greiner-Tollersrud et al first introduced the hypothesis that ADA2 might constitute a lysosomal DNase.^42^ The authors showed colocalization of ADA2 and the lysosomal protein LAMP1 by immunofluorescence staining of human tonsil samples. Moreover, they found that the glycan structures of ADA2 resembled those of key lysosomal proteins and that ADA2 could degrade DNA at an acidic pH. Testing multiple *ADA2* variants, they showed that DNase activity of ADA2 correlated with its adenosine deaminase activity. In the meantime, the authors have shown that ADA2 acts as a deoxyadenosine deaminase, altering the sequence of DNA molecules. They demonstrated that the conversion of deoxyadenosine to deoxyinosine led to increased DNA sensing by Toll-like receptor (TLR) 9, resulting in an increased type I interferon (IFN-I) response in the presence of functional ADA2.^43^ At the same time, Dong et al recently described that ADA2 bound CpG oligodeoxynucleotides in the lysosomes and thus downregulated TLR9 activation in plasmacytoid dendritic cells.^44^ While these studies looked at ADA2 from a new perspective, the modification of nucleic acids by adenosine deaminases is an old concept. In fact, the human genome encodes for a number of adenosine deaminases acting on RNA, and deleterious mutations in some of these genes cause severe inflammatory diseases.^45^^,^^46^

Considering this new angle on the function of the ADA2 protein, a thorough study of the characteristics of the intracellular protein including its processing and trafficking is warranted to then understand how defects in this protein cause the complex phenotype observed in DADA2 patients.

## ADA2 deficiency

### Clinical phenotype

In 2014, two research groups first described two independent cohorts of patients presenting with a childhood-onset phenotype of polyarteritis nodosa–like vasculitis manifesting with stroke, livedo reticularis, and systemic inflammation.^47^^,^^48^ These patients were found to carry biallelic deleterious mutations in the *ADA2* gene, resulting in a strongly reduced serum ADA2 enzyme activity.

**Fig 3:**
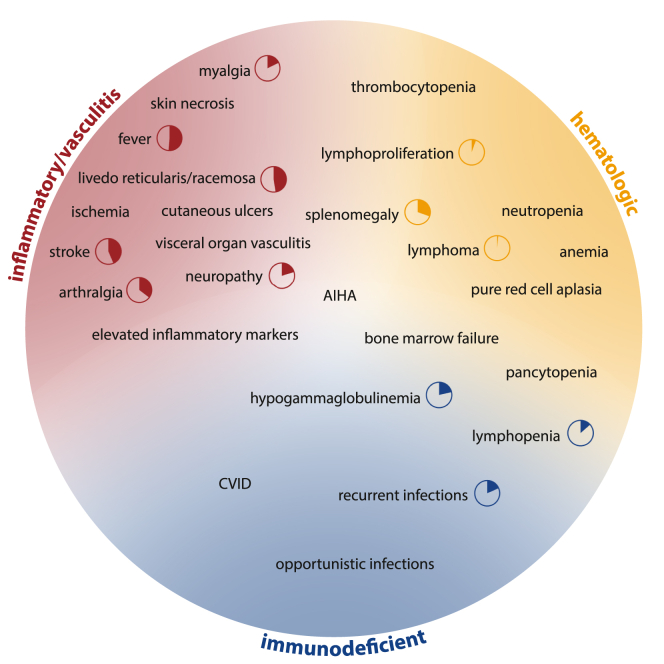
Clinical phenotype of DADA2. Depicted disease phenotypes are based on current management guidelines.^52^ Disease presents with overlapping features of different phenotypes. Prevalence of respective symptoms is indicated by pie charts where available, based on a systematic literature review published by Maccora et al.^69^*AIHA,* Autoimmune hemolytic anemia; *CVID,* common variable immunodeficiency.

While the initial clinical description focused on the vasculitic phenotype, the identification of additional patients through broad screening efforts by next-generation sequencing panels and whole-exome sequencing revealed the diversity of the clinical phenotype of DADA2.^49^ Common symptoms in the growing cohort of over 600 DADA2 patients published to date include hypogammaglobulinemia, cytopenias due to bone marrow failure, and a broad spectrum of neurologic manifestations.^18^^,^^50^ Hemophagocytic lymphohistiocytosis and malignancies represent less frequent but often life-threatening manifestations.^51^ The recently published management guidelines on DADA2 have defined 4 clinical phenotypes: (1) inflammatory/vasculitic, (2) hematologic, (3) immunodeficient, and (4) presymptomatic (ie, patients with biallelic variants in *ADA2* who are not [yet] symptomatic, perhaps identified in the context of family screening) (Fig 3).^52^ DADA2 patients often present with symptoms from different categories, but a leading phenotype can usually be identified.

Along with the expansion of the clinical phenotype, the pool of pathogenic variants in *ADA2* causing the disease has been growing. A total of 12 pathogenic variants were identified in the initial set of patients. In the meantime, 116 (likely) pathogenic variants are listed in the Infevers database.^53^ Pathogenic variants have been identified across all protein domains (Fig 1). In 2021, Jee et al calculated the estimated carrier frequency of pathogenic *ADA2* variants according to a large *in silico* analysis of the most common missense and predicted loss-of-function variants listed in gnomAD (gnomad.broadinstitute.org).^54^ On the basis of their findings, they estimated a carrier frequency of 1 in 236 individuals, resulting in a predicted prevalence of about 1 in 220,000 according to the Hardy-Weinberg equilibrium. While the majority of pathogenic *ADA2* variants causing DADA2 showed a residual ADA2 enzyme activity of <25% in their study, some DADA2-associated variants had a remnant activity up to 75% of the wild-type (WT) protein.

In the light of the great variety of pathogenic *ADA2* variants, it has been difficult to delineate genotype–phenotype associations. By analyzing 152 DADA2 patients, Lee et al proposed that *ADA2* variants showing minimal residual activity—especially nonsense and insertion/deletion variants—more frequently cause a phenotype of bone marrow failure and pure red cell aplasia, while missense variants were more prevalent in patients with a vasculitis phenotype.^55^ At the same time, there are, however, variants like p.R169Q, the most common DADA2-associated variant, that cause highly variable phenotypes in different patients, even siblings.^56^

### Immunologic phenotype

Most DADA2 patients present with some signs of systemic inflammation like fever or elevated acute-phase reactions.^57^ ADA2 protein expression is highest in monocytes and macrophages. In the absence of functional ADA2, macrophage polarization is skewed toward an M1 phenotype.^47^ Coculture with ADA2-deficient monocytes disrupts the ability of endothelial cells to form an intact cell layer.^47^

Overall, DADA2 immune cells show a proinflammatory profile. Gene expression analyses of whole blood samples from DADA2 patients revealed an elevated IFN-I signature, albeit one lower than in patients with Aicardi-Goutières syndrome, a classical type I interferonopathy.^58^ Bulk RNA sequencing and proteomic analyses of peripheral blood mononuclear cells as well as single-cell RNA sequencing of CD14^+^ monocytes confirmed the increased expression of these interferon-stimulated genes (ISGs) independently of the clinical phenotype. Moreover, these analyses showed an upregulation of type II interferon and TNF-α/NF-κB signaling.^42^^,^^59^, ^60^, ^61^ STAT1 was identified as a key regulator of the inflammatory signature. Importantly, this immune activation also remained present in patients whose disease was clinically stable while receiving treatment with TNF-α inhibitors (TNFi).^60^^,^^61^ Yet in a small cohort of 5 patients followed longitudinally there tended to be a reduction in ISG score after initiation of TNFi treatment.^62^ Like monocytes, T cells also showed an upregulation of type I and II interferon signaling by single-cell RNA sequencing.^63^ Upon stimulation of whole blood samples with TLR agonists, there was increased secretion of IL-10 and TRAIL in response to Pam_3_CSK_4_ (TLR2 agonist) and increased CXCL1 in response to ODN 2006 (TLR9 agonist) in DADA2.^47^ Stimulated ADA2^−/−^ U-937 and THP-1 cells showed increased secretion of TNF-α and IL-6.^64^^,^^65^

Besides the inflammatory phenotype, cytopenias and hypogammaglobulinemia are common symptoms of DADA2 patients. Zoccolillo et al showed that the bone marrow of DADA2 patients contained decreased numbers of stem and progenitor cells.^65^ In this cohort, this was true across different lineages, especially in myeloid, B, and erythroid cells. This is in line with experiments performed in *cecr1b*–loss-of-function zebrafish, the only currently available animal model of DADA2, which revealed the development of neutropenia in zebrafish lacking the *ADA2* ortholog.^47^^,^^66^ Defects in the differentiation of hematopoietic stem cells were recently found to be present even in samples from DADA2 patients without a hematologic phenotype, suggesting that subclinical disruption of the bone marrow might be much more common than overt bone marrow failure.^67^ In addition, DADA2 patients were found to have relatively increased numbers of transitional and naïve B cells and impaired immunoglobulin class switching.^68^ This was due to a defect in maturation while survival and proliferation were preserved. The developmental stop was also present in the bone marrow of DADA2 patients. Moreover, CD4^+^ and CD8^+^ T-cell differentiation was impaired and the cells were prone to exhaustion.^68^

### Treatment options

Many DADA2 patients described in the literature often went through several anti-inflammatory treatments before the diagnosis of DADA2 was made. These include glucocorticoids, intravenous immunoglobulins, mycophenolate, cyclophosphamide, ciclosporin A, rituximab, and biologicals targeting IL-1 and IL-6.^47^^,^^48^^,^^69^^,^^70^ Beneficial effects were occasionally reported, but most patients did not experience disease remission, and studies systematically evaluating treatment efficacy are lacking. Following the rationale that human plasma contains high concentrations of active ADA2, DADA2 patients were treated with fresh-frozen plasma in an open-label study. This approach was unsuccessful because of the rapid clearance of the ADA2 protein.^71^

The agents that have proven most effective are TNFi. They have been shown to attenuate especially the inflammatory and vasculitic symptoms and are highly effective in preventing the occurrence of stroke.^71^^,^^72^ In 2 DADA2 patients sampled before and after initiation of TNFi treatment, gene expression analysis from whole blood even displayed resolution of the inflammatory signature.^72^ Given the strong contribution of type I and II interferons to the immunologic phenotype, Janus kinase inhibitors could represent a promising additional treatment option. While these inhibitors have not been as widely applied in DADA2 as in some of the classical interferonopathies (Aicardi-Goutières syndrome, SAVI syndrome),^73^^,^^74^ case reports suggest beneficial effects of these agents in DADA2 patients as well.^51^^,^^59^

While the use of TNFi has strongly improved the outcome of DADA2 patients with a predominant vasculitis phenotype, the disease of patients presenting with immunodeficiency and bone marrow failure is often refractory to these agents. These patients require hematopoietic stem cell transplantation, the only currently available curative treatment option.^75^^,^^76^ The course of treatment is, however, often complicated by graft-versus-host disease, and despite the available therapeutic options, disease lethality of DADA2 is still 8%.

Several working groups are working on the lentiviral introduction of WT ADA2 into DADA2 patients’ hematopoietic stem cells as another curative approach.^64^^,^^65^

Development of new treatment targets would require a better understanding of the pathomechanisms driving the disease.

### Pathophysiology

In light of the complex clinical and immunologic phenotype of DADA2, researchers have not yet successfully identified a unifying pathomechanism accounting for the multiple characteristics of the disease. While pathogenic variants can occur in any domain of the ADA2 protein, they still share some features that are common to all deleterious variants identified to date: *ADA2* variants causing DADA2 exhibit reduced ADA2 activity. Patients can be identified by (close to) absent ADA2 enzyme activity in the serum. Yet the residual activity of variants associated with DADA2 when expressed in HEK293T cells varies; 91% of variants analyzed by Jee et al showed a residual activity below 25%, but there were a few pathogenic variants with a residual activity above 50% of WT ADA2 (p.F355L, p.R45W, p.G25C, p.H335P).^54^ As described above, remnant deaminase activity has been proposed to determine the phenotype of DADA2 patients, but the association is not clear-cut.^55^

Besides lacking deaminase activity, most pathogenic ADA2 variants also display impaired secretion that is associated with intracellular retention of the protein.^48^ The variance of residual secretion is great, however.^59^ The protein accumulates in the ER, and trafficking to the Golgi apparatus is impaired. The mutant protein is characterized by decreased stability and partial unfolding.^48^ Cells expressing pathogenic (missense) variants exhibit signs of an ER stress response.^59^^,^^77^ The phenotype of ER retention is also observed in variants affecting the *N*-glycosylation sites of the protein and can be reproduced by inhibiting *N*-glycosylation *in vitro.*^27^ In primary monocytes and macrophages from DADA2 patients, not only secretion but also intracellular protein expression is reduced compared to healthy control cells.^42^^,^^59^

Given the conventional function of the ADA2 protein as an adenosine deaminase, most proposed pathomechanisms involve the absence of adenosine deamination in the extracellular space. Carmona-Rivera et al found increased plasma adenosine levels in DADA2 patients.^78^ They showed that increased levels of extracellular adenosine can induce the formation of neutrophil extracellular traps (NETs). They detected NETs in a biopsy sample from a DADA2 patient and confirmed that the addition of exogenous ADA2—not ADA1—rescued NET formation induced by a surplus of extracellular adenosine. Adenosine-induced NET formation was mediated by the adenosine receptors A1 and A3. Exposure of both healthy control and DADA2 macrophages to NETs induced secretion of TNF-α, and the NET-induced increase in gene expression of the inflammatory cytokines *TNF, IL6,* and *CXCL8* was rescued by an adenosine receptor A2a antagonist. The mechanisms proposed in this paper might account for inflammatory processes in the tissue of DADA2 patients. It is unclear, however, why this allegedly adenosine-mediated mechanism is not equally attenuated by the addition of exogenous ADA1—the superior human adenosine deaminase. Moreover, another study found that A2a receptor agonists had the capacity to suppress NETosis in a model of antiphospholipid syndrome.^79^

Another mechanism by which absence of extracellular adenosine deamination could lead to an elevated inflammatory signature was proposed by Dhanwani et al.^80^ The authors found that downregulation of ADA2 expression by small interfering RNA caused an increased IFN-I response in human umbilical vein endothelial cells. They showed that this was mediated by a decrease in extracellular adenosine deaminase activity and could be rescued by the addition of either recombinant ADA1 or ADA2 to the cell culture. U-937 cells showed an upregulation of *IFNB1* gene expression in response to treatment with deoxyadenosine. The authors hypothesized that deoxyadenosine accumulated extracellularly in the absence of ADA2 and was transported to the cytosol via equilibrative nucleoside transporters. They proposed that intracellular deoxyadenosine was metabolized to deoxyinosine by ADA1, which in turn inhibited the enzyme *S*-adenosyl-methionine synthase. According to the authors, this led to increased expression of endogenous retrovirus elements due to reduced DNA methylation and therefore an upregulation of the IFN-I response.

Both studies are based on an impairment of extracellular deamination due to ADA2 deficiency. As described above, there is evidence, however, that ecto-ADA1 activity should suffice to compensate for the absence of ADA2 and nucleoside transporters allow for exchange of adenosine with the intracellular space where ADA1 is abundantly present.

An adenosine-independent mechanism has been proposed by Greiner-Tollersrud et al, who first provided evidence that ADA2 functions as a lysosomal DNase.^42^ The group revealed several similarities between ADA2 and the lysosomal protein DNase II. They suggested that the elevated IFN-I response in DADA2 was due to increased DNA sensing because of reduced lysosomal DNA degradation in the absence of functional ADA2. Indeed, they showed increased IFN-I production in an ADA2^−/−^ THP-1 cell line stimulated with interferon-stimulating DNA compared to WT-expressing cells. This was stimulator of interferon genes (STING) mediated and rescued by the lentiviral introduction of WT ADA2. Along the same lines, Dong et al showed binding of ADA2 to double-stranded DNA as well as single-stranded DNA, especially the TLR9 agonist CpG oligonucleotides.^44^ They confirmed the lysosomal localization of ADA2 and showed an increased inflammatory response due to TLR9 activation in plasmacytoid dendritic cells. Unlike the other study, the experiments performed by these researchers suggested that ADA2 protected DNA from cleavage by DNase and that binding of CpG oligonucleotides to ADA2 competed with TLR9 binding and therefore attenuated the inflammatory response. While both studies provide similar potential pathomechanisms, they are not entirely congruent. Both groups suggested increased DNA sensing leading to the elevated IFN-I response observed in DADA2. This phenotype was rescued by knockout of *STING* in the model used by Greiner-Tollersrud et al. Cyclic GMP-AMP synthase–STING signaling is, however, not classically observed downstream of TLR9. Complementing their initial work, Greiner-Tollersrud et al recently proposed that ADA2 can enhance DNA sensing by increasing the deoxyinosine content of lysosomal DNA.^43^ Deoxyinosine is classically read as deoxyguanosine by the DNA-sensing machinery. Thus, an increased deoxyinosine content in the presence of ADA2 will create CpG-like molecules and therefore facilitate TLR9 signaling, resulting in an elevated IFN-I response. Consequently, the authors showed a decreased IFN-I response in the absence of functional ADA2—a finding contrasting with the clinical phenotype and the immunologic profile of the model systems presented above. In summary, the establishment of ADA2 as a lysosomal protein/enzyme places emphasis on a potential role of ADA2 in the regulation of the IFN-I response. However, only the first 2 studies are directly in line with the increased ISG signature observed in DADA2 patients. On the one hand, if ADA2 physiologically assumed the role of attenuating IFN-I production,^42^^,^^44^ we would expect an upregulation of ISGs in the absence of functional ADA2. As a lysosomal deoxyadenosine deaminase, on the other hand, ADA2 would physiologically enhance IFN-I signaling, leading to a reduced ISG signature in ADA2-deficient cells. This cannot be directly reconciled with the actual immunologic phenotype observed in DADA2, and we would need additional mechanism accounting for the interferon-driven inflammatory profile of ADA2-deficient cells.

**Fig 4:**
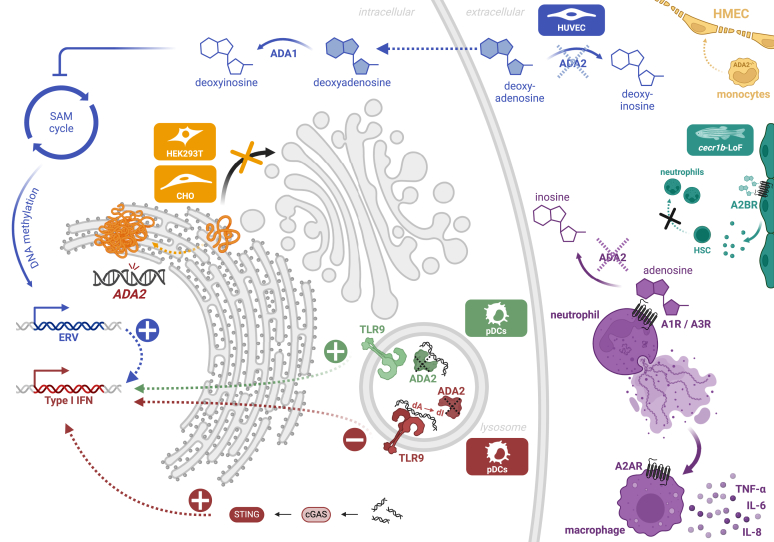
Proposed pathomechanisms driving DADA2. Several ADA2-deficient model systems have been analyzed to delineate potential cellular processes underlying DADA2.^42^, ^43^, ^44^^,^^47^^,^^59^^,^^66^^,^^77^^,^^78^^,^^80^ Different studies are distinguished by *colors. A1R,* Adenosine A1 receptor; *A2AR,* adenosine A2a receptor; *A2BR,* adenosine A2b receptor; *A3R,* adenosine A3 receptor; *dA,* deoxyadenosine; *dI,* deoxyinosine; *ERV,* endogenous retrovirus elements; *HMEC,* human microvascular endothelial cells; *HSC,* hematopoietic stem cell; *HUVEC,* human umbilical vein endothelial cells; *pDC,* plasmacytoid dendritic cell. Created with BioRender.com.

The studies listed above focus on the inflammatory phenotype of the disease and do not account for the severe cytopenias found in a large number of DADA2 patients. Recently, the zebrafish model of DADA2 was reevaluated by another group. They showed that *cecr1b*–loss-of-function zebrafish exhibited neutropenia and that colonization of the hematopoietic tissue was improved by blocking A2b adenosine receptor signaling.^66^ Thus, the authors suggested adenosine-mediated processes as the origin of the bone marrow phenotype in DADA2. As for the inflammatory phenotype, this hypothesis is limited by the enzyme kinetics of ADA1 and ADA2 presented above. An overview of the proposed mechanism driving the DADA2 phenotype on a cellular level is presented in Fig 4.

In summary, no mechanism has been identified that conclusively accounts for the clinical and immunologic characteristics of DADA2. A clear understanding of the characteristics of the ADA2 protein and its function would provide a solid foundation for the delineation of the pathophysiology, and the study of DADA2 might in turn teach us the true nonredundant function of the ADA2 protein.

## Unexplored path(omechanism)s

Here we aim to explore homeostatic pathways that are closely linked to inflammation. Many of these pathways exhibit abnormalities in the context of DADA2 and therefore represent promising candidates to explore in search of the cellular origin of the clinical phenotype.

### *N*-Glycosylation

**Fig 5:**
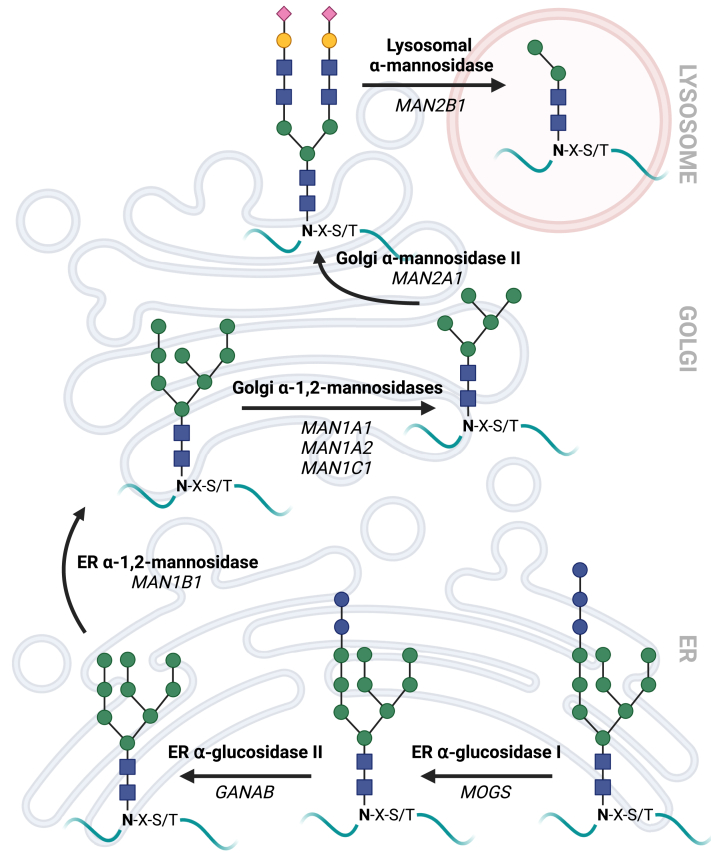
Processing of *N*-glycans. Proteins synthesized in the ER undergo glycan trimming in the ER and Golgi to mature. Removal of glycan residues also occurs in lysosomes. *Blue squares, N*-Acetyl-d-glucosamine; *green circles,*d-mannose; *yellow circles,*d-galactose; *red diamonds,* sialic acid. Created with BioRender.com.

The N-terminal signal peptide primes ADA2 for processing and trafficking via the secretory pathway.^19^ This route of protein synthesis involves multiple cellular organelles and enzymes that ensure reliable folding and transport of proteins destined for the extracellular space, the cell surface, or the inside of vesicular organelles. After protein synthesis inside the lumen of the ER, *N*-glycosylation occurs at glycosylation sites characterized by the amino acid sequence Asn-X-Ser/Thr, where X represents any amino acid except proline.^81^ Initially, glycans are synthesized onto a dolichol phosphate anchor. These glycan structures—usually containing 14 monomer units—are then transferred to the asparagine within the glycosylation site of the newly synthesized polypeptide chain.^82^ These glycan structures subsequently undergo trimming by enzymes localized in the ER and Golgi apparatus while the nascent protein is folded and trafficked to its destination (Fig 5). Glycan processing starts in the ER where α-glucosidases I (*MOGS*) and II (*GANAB*) sequentially remove glucose residues. Further trimming involves α-mannosidase I (*MAN1B1*) in the ER as well as endo-α-mannosidase (*MANEA*) and α1-2 mannosidases IA (*MAN1A1*), IB (*MAN1A2*), and IC (*MAN1C1*) in the *cis*-Golgi. Complex glycans mature later in the Golgi after cleavage by α-mannosidase II enzymes (*MAN2A1/MAN2A2*).^83^

Protein folding and *N*-glycosylation are closely intertwined. Accordingly, ADA2 variants affecting the *N*-glycosylation sites were found to cause the formation of intracellular ADA2 protein aggregates and impair ADA2 secretion.^26^^,^^27^ Besides quality control, posttranslational modifications are implicated in protein structure and function: *N*-glycans stabilize proteins and protect them from degradation in the extracellular space. Moreover, specific glycan structures are recognized by glycan-binding proteins that allow for directed transport through the cell. Glycoproteins labeled with mannose 6-phosphate are directed from the *trans*-Golgi network toward the lysosomes by binding to mannose 6-phosphate receptors.^84^^,^^85^ In fact, Greiner-Tollersrud et al performed glycan analyses and found that ADA2 is characterized by mannose-rich glycan trees and an overall glycosylation profile that was reminiscent of lysosomal proteins.^43^ Yet, Ito et al showed that secreted extracellular ADA2 contained complex glycan structures.^26^ Consequently, a thorough understanding of the glycosylation profile of ADA2 could help us define the localization and function of the protein. Recently, our group described an intracellular glycoform of ADA2 that is absent in macrophages from DADA2 patients.^86^ We showed that unlike the WT protein, mutant ADA2 does not undergo trimming by Golgi and lysosomal α-mannosidases, highlighting the potential contribution of *N*-glycosylation to the cellular DADA2 phenotype.

While the presence of glycosylation sites results from the amino acid sequence encoded in our genome, the structure of the attached glycans depends on the enzyme repertoire and the cell’s environment so that multiple glycoforms of the same protein can exist even in the same cell type. This so-called microheterogeneity of glycoproteins makes the study of protein glycosylation particularly challenging.^81^

### ER stress and the unfolded protein response

*N*-Glycosylation is part of the quality control mechanisms that ensure appropriate folding of nascent secretory proteins in the ER. Proteins that fail to undergo folding (eg, as a result of pathogenic gene variants) are recognized by UDP-glucose glycoprotein glucosyltransferase that keeps transferring α1-3-glucosyl residues onto the unfolded protein. These glycan structures are recognized by the chaperones calnexin and calreticulin that bind the respective protein to assist folding and retain the misfolded protein in the ER until the adequate conformation is attained.^81^ If folding cannot be achieved, the nascent protein will eventually be ubiquitinated and retrotranslocated to the cytosol, where it undergoes proteolysis in the proteasome, a process referred to as *ER-associated protein degradation.*^87^

**Fig 6:**
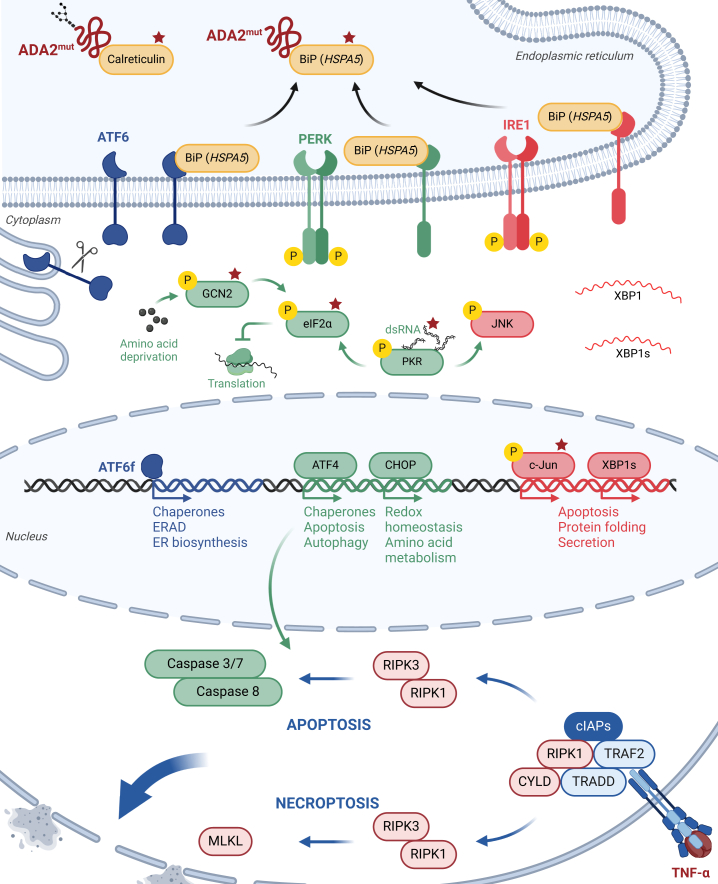
Candidate pathways driving the cellular DADA2 phenotype. *Top,* Pathways of the ER stress response have been shown to be differentially regulated in context of DADA2. Genes/proteins identified by different studies are indicated by *stars.*^59^, ^60^, ^61^^,^^63^^,^^80^*Bottom,* The RCD pathways apoptosis and necroptosis can be initiated by binding of TNF-α to TNFR1. *cIAP,* Cellular inhibitor of apoptosis protein; *IRE1,* inositol requiring enzyme 1; *MLKL,* mixed lineage kinase domain–like pseudokinase; *PERK,* protein kinase RNA-activated (PKR)-like ER kinase; *TRADD,* TNF receptor type 1–associated DEATH domain protein; *TRAF2,* TNF receptor–associated factor 2. Created with BioRender.com.

Next to calnexin and calreticulin, BiP (*HSPA5*) represents one of the major chaperones involved in ER quality control. On binding to misfolded proteins, it is released from and thereby activates the 3 major sensors of ER stress inositol requiring enzyme 1 (IRE1), protein kinase RNA-activated (PKR)-like ER kinase, and activating transcription factor (ATF) 6).^88^ Subsequent signaling via c-Jun N-terminal kinase (JNK), X-box binding protein 1 (XBP1), eukaryotic initiation factor 2α (eIF2α), ATF4 and ATF6, respectively, initiates the unfolded protein response.^89^^,^^90^ The unfolded protein response involves a number of pathways designed to maintain cellular homeostasis under conditions of increased ER stress—for example, increased synthesis of chaperone proteins, mRNA degradation to generally reduce protein synthesis, ER/Golgi biosynthesis, ER-associated protein degradation, and autophagy. If these mechanisms are insufficient to reestablish functional protein synthesis, cells exposed to ongoing ER stress will undergo apoptotic cell death.^91^ In addition, ER stress has been shown to induce an innate immune response by provoking the synthesis of major proinflammatory cytokines like TNF-α, IL-6, and IFN-Is.^92^ Consequently, pathways of the ER stress response might represent mechanisms linking the proteotype of pathogenic *ADA2* variants to the immunologic phenotype of ADA2-deficient cells. ER retention of mutant ADA2 has been shown in the HEK293T overexpression system and *HSPA5* was upregulated in peripheral blood mononuclear cells carrying pathogenic missense variants in *ADA2.*^59^ Differential expression of genes involved in the ER stress response has been shown in both DADA2 model systems and primary immune cells from DADA2 patients (Fig 6).^59^, ^60^, ^61^^,^^63^^,^^80^ This signal was, however, not uniformly present in all studied models.^77^ The different expression levels of mutant ADA2 protein in model system versus primary cells as well as the heterogeneity of glycosylation patterns are additional caveats that impede unequivocal conclusions to be drawn regarding the role of the ER stress response in the pathogenesis of DADA2.

In summary, ER stress causes a myriad of cellular processes by changing protein synthesis and secretion and by prompting inflammation and cell death. Protein misfolding due to deleterious gene variants may therefore cause a cellular and clinical phenotype driven by the unfolded protein response that is independent of the absence of the respective protein’s function.

### Cell death driving inflammation

In a cohort of 4 DADA2 patients, increased levels of spontaneous cell death were observed in CD19^+^ B cells.^47^ Moreover, as described above, protein misfolding can induce apoptotic cell death.^91^ In recent years, the definition of different forms of cell death and the increasing understanding of the underlying pathways have uncovered the relevance of cell death regulation in the context of inflammation. Regulated cell death (RCD)—as opposed to accidental cell death—is characterized by its controllability by components of the respective signaling pathways and thus accessibility for pharmaceutical inhibitors.^93^^,^^94^ Major forms of RCD include apoptosis, necroptosis, and pyroptosis. They are all clearly defined by the involvement of key molecules and responsiveness to specific inhibitors. *In vivo,* the different pathways may overlap, however, and the driving form is not always distinguishable.

Apoptosis is known as the classic form of programmed cell death, a form of cellular suicide that can occur physiologically without any cellular imbalance. It is, however, also induced by intracellular stress signals or via surface receptors.^95^ Intrinsic apoptosis is initiated by the permeabilization of the mitochondrial outer membrane and mediated by proteins of the BCL2 family. It eventually results in activation of caspase (CASP) 9 that in turn activates the executioner caspases CASP3 and CASP7 by proteolytic cleavage. However, extrinsic apoptosis starts on binding of ligands to death receptors like Fas cell surface death receptor (FAS) or TNF receptor 1 (TNFR1) on the cell surface. Downstream, this leads to the activation of CASP8 followed by the activation of CASP3 and CASP7 for the intrinsic pathway.^96^

Like extrinsic apoptosis, necroptosis can equally be triggered by binding of ligands to cell surface receptors like FAS and TNFR1, but also intracellular receptors like TLR3 and RIG-I–like receptors. The key mediators of necroptosis are receptor-interacting serine/threonine-protein kinase (RIPK) 1, RIPK3, and mixed lineage kinase domain-like pseudokinase (MLKL) that are subsequently phosphorylated after initiation of the necroptosis pathway. Phosphorylation of MLKL finally leads to oligomerization of the protein and membrane permeabilization by the protein complex.^97^

The overlap of the signaling pathways of extrinsic apoptosis and necroptosis highlights the interconnectivity of the different pathways and points to the challenge of studying the physiologic pathways *in vitro*. The same trigger can start either pathway depending on the activity of CASP8.^97^ Importantly, both RCD pathways can be initiated by binding of TNF-α to TNFR1. In the light of the clinical efficacy of TNFi in the treatment of DADA2, apoptosis and necroptosis might therefore represent the most likely candidates accounting for the elevated cell death levels observed in ADA2-deficient cells.^47^

Pyroptosis was identified as a proinflammatory form of RCD.^98^ The process is initiated by sensing of danger signals by inflammasomes that cause caspase activation. Canonical pyroptosis is mediated by CASP1, but CASP3, CASP4, and CASP5 have also been shown to drive pyroptosis.^99^^,^^100^ Active caspases cleave gasdermins to form pores in the plasma membrane, prompting pyroptotic cell death. CASP1 also generates mature IL-1β and IL-18, the secretion of which is classically associated with pyroptosis.

In addition, PANoptosis represents a form of cell death that unites features of pyroptosis, apoptosis, and necroptosis and is mediated by the Z-DNA binding protein 1 (ZBP1) that senses intracellular nucleic acids. Interestingly, ADAR1—an RNA-specific human adenosine deaminase—negatively regulates PANoptosis in a ZBP1-dependent way.^101^

All these processes can either be triggered by inflammatory stimuli or can propel inflammation by the release of danger signals. Observing increased cell death in inflammatory diseases therefore usually confronts the researcher with a classical chicken-or-egg conundrum. Likewise, in DADA2, we can speculate whether the inflammatory signature of ADA2-deficient cells is responsible for the increased propensity for cell death or whether increased cell death—for example, as a result of homeostatic dysbalances in the absence of functional ADA2—contributes to the immunologic phenotype of the disease.

In addition, a deeper understanding of the involvement of RCD in the pathogenesis of a disease can lay the foundation for new treatment options. The implementation of IL-1β inhibition as a treatment strategy has greatly improved the outcome of patients with familial Mediterranean fever, a disease characterized by increased levels of pyroptosis.^102^^,^^103^ Mutations in *RIPK1* and *TBK1* cause aberrant necroptosis manifesting with an inflammatory phenotype.^104^, ^105^, ^106^ Necroptosis can be induced by stimulation with TNF-α in the presence of inhibitors of inhibitor of apoptosis proteins and caspases. Indeed, treatment with TNFi improved the clinical symptoms of some of the affected patients.^104^

In summary, the definition of the drivers of cell death in DADA2 may pave the way for the identification of targeted treatment options.

## Conclusion

The clinical management of DADA2 remains challenging as a result of the complexity of the disease and the limited understanding of the underlying pathomechanisms. There is an unmet need for alternative treatment options, especially for patients with bone marrow failure, whose disease is often refractory to TNFi. Ideally, we should strive for a more personalized—potentially genotype or phenotype dependent—therapeutic approach to avoid long periods of unsuccessful treatment attempts that might result in frequent disease flares or complications and worsen long-term outcomes.

The search for new treatment targets is impeded by the uncertainty about the characteristics and function of the ADA2 protein. The relevance of the protein’s deaminase activity in the pathophysiology of the disease has been debated. Without identifying the physiologically relevant function of the ADA2 protein, it will remain difficult to determine the disease-causing effects of pathogenic variants in *ADA2* and—in a second step—establish the differences between the various genotypes. The study of DADA2 therefore requires a thorough approach uniting the subcellular, immunologic, and clinical aspects of the disease.

## Disclosure statement

Supported by the European Research Council under the European Union’s Horizon 2020 research and innovation program (GA no. 948959; MORE2ADA2). I.M. is a senior clinical investigator at the Research Foundation–Flanders and is supported by a KU Leuven C1 grant (grant C16/18/007); by the Research Foundation–Flanders (FWO; grant G0B5120N); and by the Jeffrey Modell Foundation. This work was supported by ERN-RITA. L.E. was supported by a PhD fellowship from FWO (grant 11E0123N). L.E. is a fellow of the BIH Charité Junior Clinician Scientist Program, funded by the Charité–Universitätsmedizin Berlin, and the Berlin Institute of Health at Charité.

Disclosure of potential conflict of interest: I. Meyts declares support from KU Leuven Research and Development; receipt of advisory board honoraria from Takeda and Boehringer-Ingelheim; and receipt of research funding from CSL-Behring through a chairship at KU Leuven. The other author declares no relevant conflicts of interest.
